# Examining the links between temperature, piglet behaviour and survival during winter in outdoor housing in Australia

**DOI:** 10.1017/awf.2026.10069

**Published:** 2026-02-10

**Authors:** Amelia H Sofra, Lauren M Hemsworth, Megan E Lucas

**Affiliations:** Animal Welfare Science Centre, Faculty of Science, The University of Melbourne, Parkville, VIC 3010, Australia

**Keywords:** Animal welfare, cold stress, free-range, piglet overlays, pre-weaning mortality, thermal comfort

## Abstract

Cold stress is a significant welfare concern for piglets, particularly in outdoor housing systems where the ambient climate cannot be controlled. To deal with cold stress, piglets engage in heat-inducing behaviours, such as maintaining proximity to the sow, however this is a major risk factor for overlays. This research examined the effect of outdoor farrowing hut temperature on piglet survival and behaviour and found that lowered hut temperatures led to increased pre-weaning mortality. Two hundred sows and their litters were studied over six time replicates during winter in Australia at a commercial outdoor piggery. As daily minimum hut temperature decreased, piglet mortality increased on the first day of life, and across days one to four of life, but not from five days onwards. As hut temperature decreased, piglets were more likely to be shivering, huddling in one group, and resting in physical contact with the sow. However, contrary to expectations, there was no evidence of a relationship between piglet-sow proximity and piglet deaths, suggesting that being in close proximity to the sow did not increase the risk of dying. It may be that cold exposure reduces piglet mobility and thus the chance for piglets to avoid being crushed, but this requires further examination. Overall, cold stress is clearly a significant piglet welfare and productivity concern leading to increased mortality even when considering more moderate Australian climates.

## Introduction

A variety of housing systems are used in the pork industry, including outdoor housing where pigs (*Sus scrofa*) are kept outside with access to shelter during all stages of production. While features vary depending on locations and certification schemes, outdoor farrowing systems typically consist of paddocks with rooting areas, wallows where permitting, and huts for shelter with deep straw bedding. Pigs housed in outdoor farrowing systems show a range of behaviours associated with positive welfare, such as exploratory, foraging and play behaviours, and demonstrate fewer agonistic interactions and abnormal behaviours, such as sham-chewing in sows and belly-nosing in piglets, than pigs housed in conventional indoor systems (Hötzel *et al.* 2004; Tozawa *et al.* 2016).

Although outdoor farrowing systems offer several benefits to pigs, there are also a range of welfare concerns associated with this type of housing. Overlaying is the crushing of a piglet by the sow, generally during postural changes like lying down or rolling (Danholt *et al.* 2011), and accounts for around three-quarters of pre-weaning mortality in outdoor systems, mostly within the first four days of life (Edwards *et al.* 1994; Marchant *et al.* 2000; Rangstrup-Christensen *et al.* 2018a). Overlays also occur in conventional indoor systems but are exacerbated in outdoor systems where the sow is not contained. A number of hut designs and features have been examined with the aim of reducing overlays, and while there have been some improvements, overlays continue to be a major animal welfare concern (Rangstrup-Christensen *et al.* 2018a; Kobek-Kjeldager *et al.* 2023). Overlays also have consequences for farm performance, which may disincentivise producers from utilising outdoor systems despite the animal welfare benefits they offer (Baxter *et al.* 2012).

One factor likely to increase overlays in outdoor farrowing systems is cold weather. This is because piglets are most susceptible to being overlain when in close proximity to the sow, defined as “the danger zone” (Pokorná *et al.* 2008), and piglets are more likely to be in this danger zone seeking heat from the sow in cold conditions (Weary *et al.* 1996). As piglets do not have brown adipose tissue, a typical thermogenic tissue in mammals, they instead rely almost entirely on behavioural adaptation and shivering thermogenesis early in life, mobilising their minimal fat and glycogen reserves (Lossec *et al.* 1998; Mrowka & Reuter 2016). In the first few days of life, piglets have a considerably high thermoneutral zone of around 27 to 35°C, and due to their limited thermoregulatory abilities are particularly vulnerable to cold stress at this time (Herpin *et al.* 2002; Villanueva-García *et al.* 2020). In outdoor systems, deep straw bedding helps with thermoregulation by reducing heat loss to the floor (Algers & Jensen 1990), but it is not typical to provide supplementary heat sources like heat lamps or mats. This exacerbates the need for piglets to rely upon using their behaviour to thermoregulate. In addition to seeking physical contact from the sow, piglets also demonstrate thermoregulatory behaviours such as shivering and huddling with one another to reduce heat loss to the environment. Therefore, optimising the microclimate inside the farrowing hut is particularly crucial to piglet welfare early in life, as suboptimal temperatures inside the hut can lead to hypothermia and increase the risk of overlay if more piglets are in the danger zone.

Previous research has examined the direct consequences of cold stress in outdoor systems, such as hypothermia and metabolic studies (Gade 2002; Kammersgaard *et al.* 2011; Vandresen *et al.* 2021) but less attention has been paid to how low hut temperature affects piglet thermoregulatory behaviours and survival. Compared to the UK and Europe, there has been minimal research in an Australian context, where the temperature is more moderate over winter. This research aimed to examine links between farrowing hut temperature, piglet behaviour, and piglet survival during winter in Australia, under typical outdoor pig farming conditions. It was hypothesised that as hut temperature decreased, piglets would engage in more thermoregulatory behaviours including spending more time in contact with the sow, which would lead to increased piglet deaths, potentially caused from sow overlay.

## Materials and methods

This research was conducted in winter from June 2024 to August 2024, on a commercial outdoor piggery in southern Victoria, Australia.

### Ethical considerations

Ethical approval for this project was granted by an Animal Ethics Committee at the University of Melbourne, Australia (approval ID: 29590). The research was conducted in accordance with the Australian Code for the Responsible Care and Use of Animals for Scientific Purposes.

### Study animals, housing and experimental design

A total of 200 Landrace × Large White × Duroc or Landrace × Large White sows and their litters were studied for 12 days post-farrowing, over six consecutive time replicates. There were five primiparous sows, 124 sows of parity 2–4, and 71 of parity 5–10. All sows were artificially inseminated apart from eight in one replicate that had been mated naturally. Approximately one week prior to expected parturition, sows were moved from an outdoor gestation unit to group farrowing and lactation paddocks, with each paddock containing eight sows and eight farrowing huts. Sows were free to move around the paddock and choose a farrowing hut to perform nest-building behaviours. Either four (Replicate 1, 2, 4, 5 and 6) or five (Replicate 3) paddocks were studied in each replicate (n = 32 or n = 40 sows and their litters per replicate). There was a farrowing spread of 6–9 days between litters within the same replicate, and 1–2 weeks between farrowings for consecutive time replicates. Each paddock contained one water source, one feeder, and a wallow.

**Figure 1.**
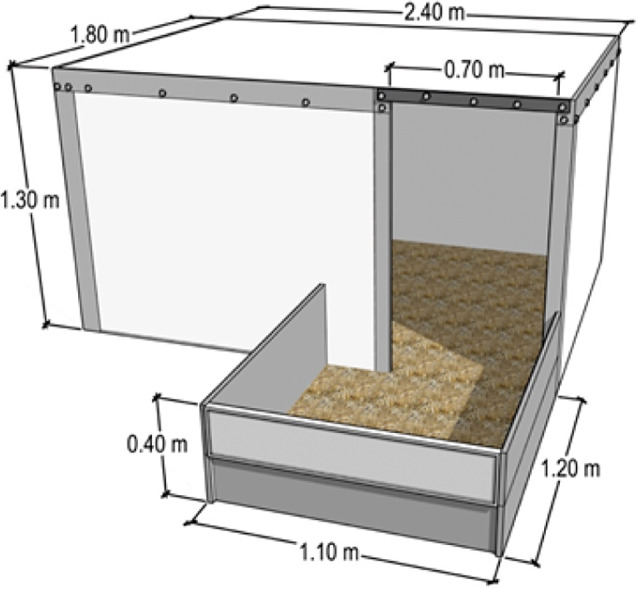
Diagram of the outdoor farrowing and lactation hut pigs were housed in for the experiment, with the outdoor area pictured. Half of the huts in each paddock also contained a transparent door covering over the entryway (not pictured).

Each hut was designed to house one sow and her litter. The huts measured 2.4 × 1.8 × 1.3 m (length × width × height) and contained one entryway (1.3 × 0.7 m; length × width), one small window (either 0.6 × 0.6 m or 0.6 × 0.3 m) that was covered throughout the experiment, and a small outdoor area contained by a low metal fence (1.2 × 1.1 m; Figure 1). For the first 2 weeks post-farrowing, the fence was 0.4 m in height, a level that allowed the sow to step over the fence to access the rest of the paddock, but limited piglets from leaving the hut and small outdoor area early in life (Figure 1). After two weeks, a portion of the front of the fence was removed, lowering the height and allowing piglets full access to the paddock and other litters. The huts were constructed from metal and did not contain any supplementary heat sources. While they were insulated, some were relatively old, and it was not possible to determine whether insulation was fully present or partially degraded. The present research was conducted within a larger project examining the effect of an intervention, a door flap covering the entry way of pigs’ shelter, in reducing heat loss and piglet mortality (Lucas *et al.* 2025). For this larger research project, a randomised complete block design with paddock as the blocking factor was used to assign treatments (with or without door flap) to huts. Thus, within the present research, half of the huts in each paddock contained a transparent rubber door flap that covered three-quarters of the hut entryway. All huts were positioned with the entryway facing north to north-east.

Pigs were managed by stockpeople according to typical commercial practice, which included at least one daily visual inspection of all animals. An abundant amount of straw (roughly 2–3 bales per hut) was provided inside the hut prior to farrowing and was replenished as needed, based upon the discretion of stockpeople. Feed was provided *ad libitum* to sows, and additional feed was dropped into the small outdoor area once per day for the first few days post-farrowing. Within 24 h of farrowing (day 0), stockpeople undertook routine postnatal litter management, which involved cross-fostering to equalise the weight of piglets within litters and the number of piglets across litters. Stockpeople assigned each litter to a weight category of ‘Small’, ‘Medium’ or ‘Big’, based on visual inspection of piglet sizes during fostering. Fostering was conducted within the two treatments (huts with or without door flap) being examined in the larger research project. During this process, some female piglets that were selected as breeding stock were ear-notched for identification and had half of their tail docked, while the remaining piglets did not undergo any husbandry procedures. In addition to routine management by stockpeople, the researchers recorded in-person piglet behaviour, straw temperature, straw moisture content, and ammonia concentrations for each hut once a day from 0 to 4 days of age, for the purposes of the larger research project examining the impact of door flaps as reported in Lucas *et al.* (2025). Piglets were weaned at a mean (± SD) age of 26 (± 2.7) days.

### Measurements

#### Behavioural observations

Video observations were recorded, downloaded and then analysed retrospectively by one trained researcher for a subset of litters (n = 59; control huts = 26, door flap huts = 33). Prior to farrowing, Swift 3C scouting trail cameras (Outdoor Cameras Australia, Toowoomba, Australia) were placed in ten huts per replicate. One camera failed, and three were placed in door flaps instead of control huts, leading to a slightly greater representation of litters from door-flap huts. Each camera was set to record for 5 s every 10 min. Instantaneous scan sampling was used to record various piglet and sow behaviours, including piglet proximity to the sow when both were resting.

**Figure 2.**
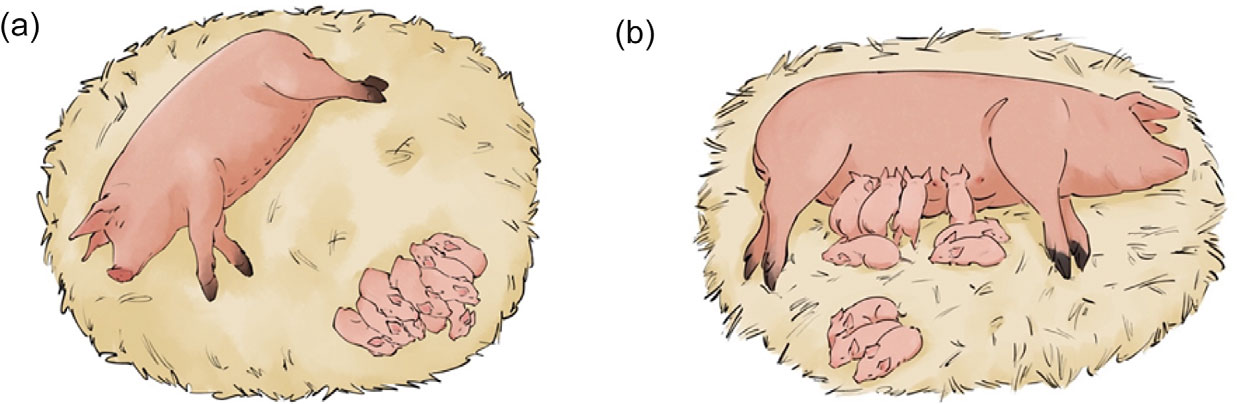
Shows example sows and litters with piglets at various proximities. The litter in diagram (a) has all piglets resting in a huddle greater than one piglet length away, and this litter is eligible for observation as the sow and all piglets are resting. The litter in diagram (b) has one group of piglets awake at udder and in contact, one group resting and < 1 piglet length away and one group resting and > 1 length away. The piglets are observed in separate groups and thus are not huddling. This litter is ineligible for observation, as some piglets are attempting to nurse, and thus less than 80% of the litter are resting.

As one of the aims of the present research was to examine if piglets spend more time resting in close proximity to the sow in cold conditions, behavioural observations of sows and litters were recorded when both were simultaneously lying stationary, without active or attempted nursing by the piglets. The litter was considered to be resting when at least 80% of piglets were lying stationary anywhere within the hut or the outdoor area, typically with their eyes closed, and not in oral contact with the sow’s udder. When both the sow and piglets were resting, piglet proximity to the sow was recorded by counting the number of piglets in contact with the sow, within one piglet length of the sow, more than one piglet length from the sow, and out of view of the camera (Figure 2). Whether or not the majority of piglets (>50%) in the litter were shivering, and whether or not the litter was lying a single huddle (see Figure 2[a]) was also recorded. These behaviours were recorded every hour from 1200 to 2300h from day 0 to 4 (a total of 60 attempted observations per hut). The first recorded clip after 1200h was observed, and so on every hour until 23000h. This window of observation was chosen as it was after routine stockperson observation and intervention in the morning and allowed for observations over a larger range of temperatures and sunlight. When observations could not be taken (e.g. sows/piglets were active), the sampling interval was skipped.

#### Hut temperature

**Figure 3.**
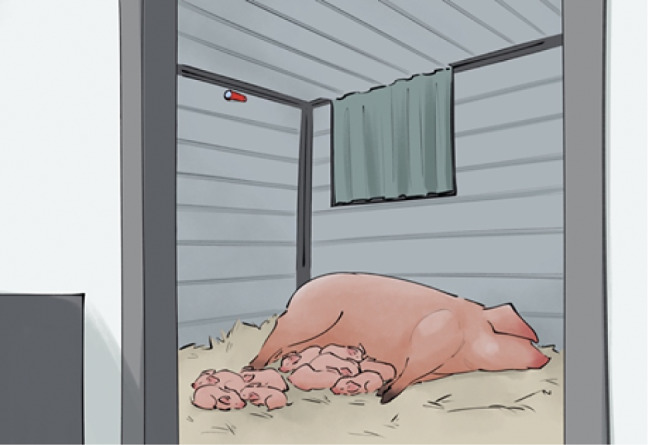
The inside of the outdoor farrowing and lactation hut viewed from the entryway, with a sow and litter inside. The red temperature logger can be seen at the midpoint of the back wall of the hut.

Prior to farrowing, a temperature logger was placed inside each hut (either a Thermochron TC Temperature Logger DS1921G or Hygrochron Humidity/Temperature Logger DS1923, both Thermochron, Castle Hill, Australia). The temperature loggers were positioned on the top of the midpoint of the short wall opposite the hut entryway (Figure 3), and recorded hut temperature every hour. The loggers measured temperature with a precision of 0.5°C. Data were downloaded retrospectively by the researchers when the temperature loggers were removed at 12 days of age to allow re-use in other huts.

#### Piglet survival

Stockpeople at the farm recorded information on piglet survival for each litter until weaning. This included the number of piglets born alive, stillborn, mummified, and weaned, and the timing and cause of piglet mortality. Causes of piglet mortality at litter management on day 0 of age included: overlain by sow; overlain by sow who farrowed outside the hut; exposure (e.g. hypothermia/chilling); attacked by predator/missing; or other/unknown. Causes of piglet mortality post-litter management on day 0 until weaning included: overlain by sow; exposure; missing, predation or savaged by the sow; and unknown. The number of piglets alive in the litter was also recorded by the researchers every day from 0 to 12 days of age to cross reference against mortality records for completeness.

### Statistical analysis

All data were analysed in RStudio, Version 2024.04.2+764 (R Core Team 2024) by fitting either a Linear Mixed Effects Model or a Generalised Linear Mixed Model using the packages lme4 (Bates *et al.* 2015), lmerTest (Kuznetsova *et al.* 2017) and glmmTMB (Brooks *et al.* 2017). The experimental unit was always the litter of pigs. For all Linear Mixed Effects Models, model fit was examined by checking for outliers, normality and homoscedasticity by visually inspecting the residual plots. For all Generalised Linear Mixed Models, a Pearson Chi-squared test was performed to test for overdispersion. Odds ratios for these models are reported as back-transformed values calculated from exponentiating the model estimates. Pairwise comparisons were conducted using the pairs function of the emmeans package (Lenth *et al.* 2025).

Where relevant, models included the following fixed effects: day of piglet age (day 0, 1, 2, 3 or 4, with day 0 representing the first day of life); the litter’s weight category (small, medium or big, based on categorisation by the stockperson on day 0 as described previously); the time of day to the closest hour (1200, 1300 and so on until 2300h) and sow parity (parity 1, parity 2–4 or parity 5–10). To account for the hierarchical structure of the data, random effects for hut, paddock and replicate were included, with huts nested within paddocks which were nested within replicates, as well as day of age nested within the others for models examining piglet behaviour. Further detail pertaining to specific variables and each of the model structures is presented below.

### Effect of hut temperature on piglet mortality




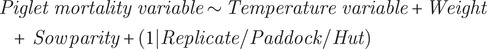

Generalised Linear Mixed Models using a binomial distribution with a logit-link function were fitted to examine the effect of hut temperature on piglet mortality. For analyses of piglet survival, the hut temperature variables included the average minimum and average hut temperature during three time-periods that corresponded to the piglet mortality variables: day 0, days 1 to 4, and days 5 to 12. Minimum and average temperature variables for these periods were calculated from averaging the daily minimum temperature over these days, and averaging the hourly hut temperature each day, respectively. Piglet mortality variables after litter management on day 0 included the number of piglet deaths due to any cause from days 1 to 4 and 5 to 12. For analyses of piglet survival before litter management on day 0, the piglet mortality variable was the total number of piglet deaths on day 0 for all sows who farrowed in a hut.

While specific causes of death were recorded by stockpeople according to routine practice on-farm, overwhelmingly most deaths had the recorded cause of overlay. For instance, on day 0, 100% of all piglet deaths inside a hut were recorded as overlay, with no deaths listed as being caused by starvation and only two who farrowed outside by hypothermia. It is likely that overlay was a secondary cause of death in some of these cases (i.e. the piglet died of starvation and was subsequently overlain). As such, it was decided to analyse piglet mortality by looking at all causes of death together as there was a degree of uncertainty regarding the accuracy of mortality causes.

Sow parity was included as a fixed effect in these models as previous studies have shown that sow parity can be a risk factor for increased mortality (Lossec *et al.* 1998; Galiot *et al.* 2018). Days 1 to 4 were chosen specifically to look at the number of piglets that died early in life, as most piglet mortality occurs before this time (Svendsen *et al.* 1986; Marchant *et al.* 2001; Tucker *et al.* 2021), while days 5 to 12 were the remaining days in which piglets were still confined to the hut and hut temperature measurements were collected. In all piglet mortality models, the number of piglet deaths were compared to the number of piglets that had been alive in the litter at the start of the time-period; either the number born alive, or number alive on day 1 or day 5. In these cases, the number of piglets that had died on day 0 was compared to the number born alive. For piglet mortality on day 0, the litter’s weight classification was not included as a fixed effect as piglets had not yet been fostered and thus were of different weights.

### Effect of temperature on piglet-sow proximity




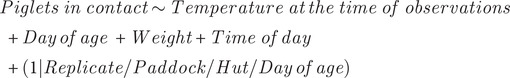

Generalised Linear Mixed Effects Models with a beta-binomial distribution were fitted to examine how hut temperature and day of age affected the proportion of the litter observed in physical contact with the sow from days 0 to 4 of age. Time of day was included in this model to control for systematic variation that may influence behaviour from day to night, such as influences of diurnal behaviour and presence of humans. An interaction between hut temperature at the time of observation and day of age was fitted, but there were no significant (*P* < 0.05) interactions, thus it was dropped, and odds ratios were reported from the additive model. While piglet behaviours were quantified based on the number of piglets resting in contact with the sow, within one piglet length of the sow, and more than one piglet length from the sow, the direction of effects for these different behaviour variables was similar. Thus, for conciseness, only statistical modelling for piglets resting in contact with the sow is reported. Temperature was quantified as the temperature of the hut at the closest hourly time-point to behavioural observations of piglet-sow proximity.

### Effect of piglet-sow proximity on piglet mortality




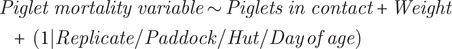

Generalised Linear Mixed Models using a binomial distribution with a logit-link function were fitted to examine the effect of piglet-sow proximity on piglet mortality. One piglet mortality variable was the total number of piglet deaths from days 1 to 4. For this model, average values for piglets in contact with the sow were calculated from the proportion of piglets in physical contact with the sow when both the sow and piglets were resting from 1200 to 2300h across days 1–4. Mortality and proximity over day 0 was also examined, where the piglet mortality variable was the number of piglet deaths on day 0 post-litter management, and the proximity variable was the resting behaviour of piglets from 1200 to 2300h.

### Effect of temperature on shivering and huddling behaviours




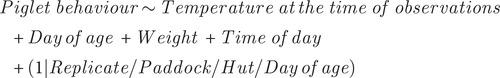

Generalised Linear Mixed Models using a binomial distribution with a logit-link function were fitted to examine the effects of hut temperature on piglet thermoregulatory behaviours each hour. Piglet thermoregulatory behaviours included binary variables for whether most piglets in the litter were shivering (yes or no), and whether the litter was huddling as one group (yes or no) when resting. Temperature was quantified as the temperature of the hut at the closest hourly time-point to behavioural observations.

### Number of huts and observations included in each model

A total of 200 sows and their litters were included in the study, but not every model contained a complete dataset from each of these 200 litters. Similarly, not all 59 huts that had cameras in them contained a full set of observations. A full dataset of 200 litters or 3,540 behaviour/temperature observations were not always present due to several reasons: four temperature loggers malfunctioned, weight category was not assigned to two litters, and piglet behavioural observations were unable to be completed if the sow or piglets were feeding or active. Further, when analysing piglet survival, there were 14 sows that farrowed outside of a hut or in another occupied hut, which were excluded from piglet mortality on day 0 and behavioural observations, as any piglet deaths that occurred at this time were not affected by hut temperature. The eleven sows that farrowed outside were moved into a farrowing hut as soon as possible, and as it was only a small number of huts, they were included in analyses of piglet mortality and temperature but excluded from any behaviour or mortality models that included observations on day 0.

## Results

Throughout the study period from early winter until early spring, temperatures inside of the farrowing huts ranged from –1 to 29°C over days 0 to 12 of life for piglets. During this time, ambient weather conditions at the nearest weather station ranged from 1.5 to 22.2°C. The median average daily hut temperature was 14.7°C, and the median minimum daily hut temperature was 11°C.

**Figure 4.**
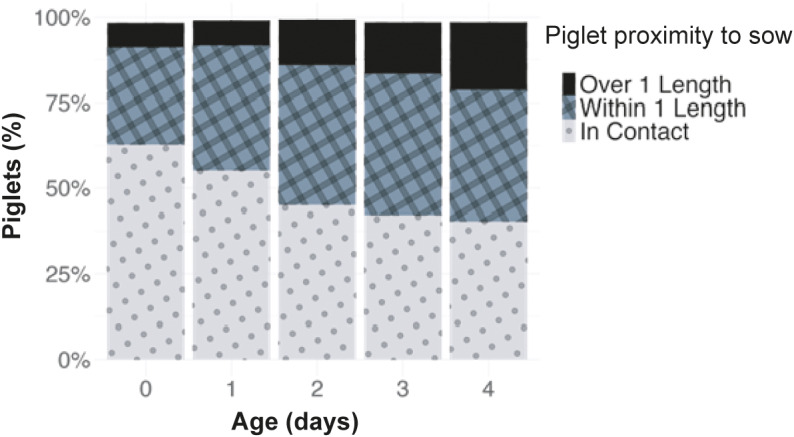
Descriptive data on the percentage of piglets resting in contact with the sow (dotted bars), less than one piglet length from the sow but not in contact (crosshatched bars), and more than one piglet length away from the sow (solid bars).

A mean (± SD) of 12.2 (± 3.2) piglets were born alive per litter; average litter size post-litter management was 10.9 (± 1.7) and 9.6 piglets (± 1.85) per litter were weaned. From all piglets that were recorded alive by stockpeople on day 0, 5% died by day 1, 7% died by day 2, 8% died by day 3 and 10% died by day 4. Of all piglets born alive, 17% died before weaning. Over half of the piglets were in contact with the sow on day 0, which decreased with time, and the proportion less than or greater than one piglet length from the sow increased (Figure 4).

### Piglet survival and hut temperature

Overall, there was evidence that hut temperatures were associated with early life piglet mortality, in the direction of colder huts leading to more piglet deaths (*P* < 0.05; Table 1). For every 1°C increase in minimum hut temperature on the day of farrowing, the odds of piglets dying from any cause in the first 24 h of life decreased by 11% (*P* = 0.044). Similarly, for every 1°C increase in minimum hut temperature from days 1 to 4 of age, the odds of piglets dying from any cause decreased by 13% (*P* = 0.009). There was no further effect of hut temperature on piglet mortality after four days of age. Additionally, only minimum hut temperature rather than average hut temperature had a significant effect on piglet mortality on day 0 and over days 1 to 4 (*P* > 0.05).

**Table 1.** Effects of mean daily minimum and average hut temperatures on the number of piglet deaths before litter management on day 0, from days 1 to 4, and 5 to 12 of life

Piglets	N _Hut_	OR [95% CI]	*P*-value
Died from any cause before litter management on day 0			
Minimum hut temperature on day 0 of age	180	0.89 [0.80–1.00]	**0.044**
Average hut temperature on day 0 of age	180	0.92 [0.80–1.06]	0.255
Died from any cause from days 1 to 4			
Minimum hut temperature from days 1 to 4 of age	193	0.87 [0.78–0.97]	**0.009**
Average hut temperature from days 1 to 4 of age	193	0.89 [0.78–1.02]	0.095
Died from any cause from days 5 to 12			
Minimum hut temperature from 5 to 12 days of age	193	1.21 [0.98–1.50]	0.073
Average hut temperature from 5 to 12 days of age	193	1.18 [0.97–1.45]	0.100

OR = Odds ratio. Values in bold are statistically significant at *P* < 0.05.

There was strong evidence that piglets from litters classified as ‘Small’ on day 0 were more likely to die across days 1 to 4 (Table 2). For models that fitted the effect of minimum hut temperature on piglet survival, the odds of piglets from ‘Big’ litters dying were 68% lower than piglets from ‘Small’ litters (Table 2). There was no difference in the odds of survival of ‘Medium’ and ‘Big’ piglets (*P* > 0.05 for temperature and mortality over 1 to 4 days of life). For models that fitted the effect of average hut temperature on piglet survival over days 1 to 4, the direction and strength of the effect of weight was essentially identical. There was no effect of piglet weight category on piglet survival on over days 5 to 12 of life.

**Table 2.** Effects of piglet weight category assigned on day 0 (small, medium or big) on piglet survival from days 1 to 4 for models fitting minimum and average hut temperature as predictors

Model	Litter weight	Deaths from days 1 to 4	
		OR [95% CI]	*P*-value
Minimum hut temperature	Small	1	-
	Medium	0.44 [0.18–1.07]	0.069
	Big	0.32 [0.15–0.70]	**0.004**
Average hut temperature	Small	1	-
	Medium	0.42 [0.17–1.05]	0.064
	Big	0.31 [0.14–0.68]	**0.004**

OR = Odds ratio. Values in bold are statistically significant at *P* < 0.05.

There was no detected effect of sow parity on the number of piglets that died on day 0 or from days 5 to 12 (*P* > 0.05). However, during days 1 to 4 there was evidence that piglets from parity 1 sows were more likely to die compared to those from parity 2–4 and parity 5+ sows (Table 3). As with the effects of weight, the direction and strength of all parity effects on piglet survival after day 0 were similar for models that fitted average and minimum hut temperature.

**Table 3.** Effects of sow parity category (parity 1, parity 2-4 or parity 5+) on piglet survival from days 1 to 4 for models fitting minimum and average hut temperature as predictors

Model	Sow parity	Deaths from days 1 to 4	
		OR [95% CI]	*P*-value
Minimum hut temperature	Parity 1	1	-
	Parity 2–4	0.17 (0.05–0.58)	**0.004**
	Parity 5+	0.28 (0.08–0.94)	**0.039**
Average hut temperature	Parity 1	1	-
	Parity 2–4	0.18 (0.05–0.61)	**0.006**
	Parity 5+	0.29 (0.08–0.99)	**0.048**

OR = Odds ratio. Values in bold are statistically significant at *P* < 0.05.

### Piglet-sow proximity and hut temperature

**Figure 5.**
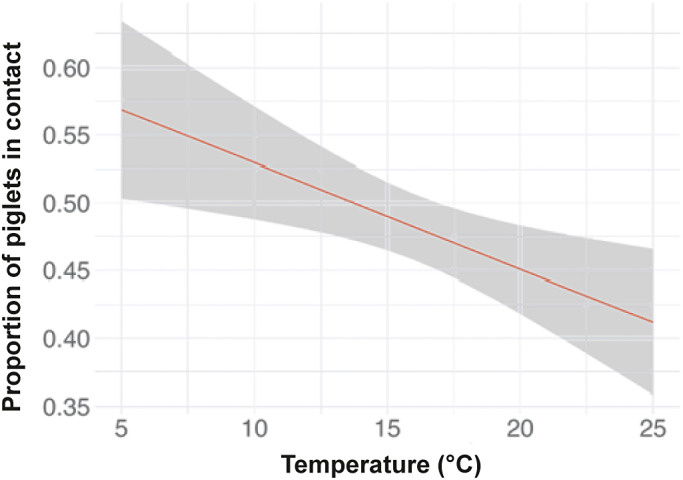
Relationship between hut temperature and the proportion of piglets in contact with the sow when resting early in life. Data are combined for days 0 to 4 of age. Piglet-sow proximity and hut temperature were measured at a similar point in time. Grey shading represents the 95% CI. N_Litters_ = 59, N_Behaviour observations_ = 2,175.

**Figure 6.**
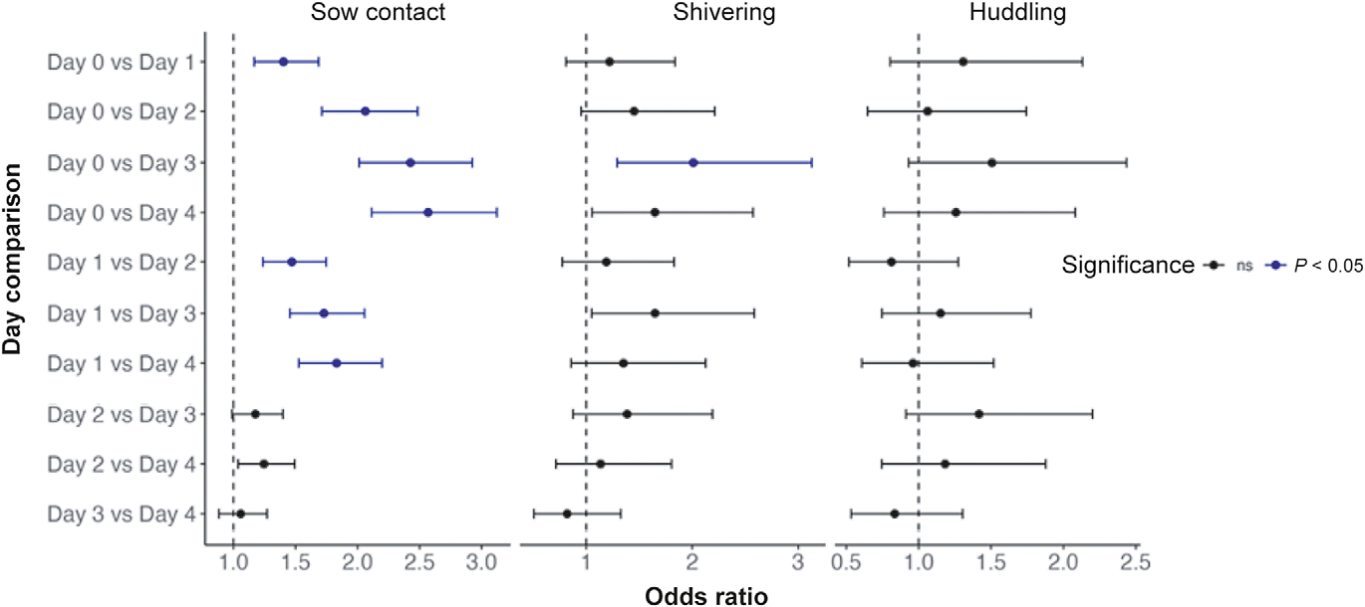
Odds ratios with 95% confidence intervals for the likelihood of piglets resting in contact with the sow, huddling in one group, and shivering, across day 0 to 4 of life. Each point represents the odds ratios for a specific day comparison. Points to the right of the dashed line at odds ratio = 1, indicate the behaviour is more likely on the first day listed compared to the second, with significant differences (*P* < 0.05) highlighted in blue.

There was a significant negative relationship between hut temperature and the proportion of piglets in contact with the sow at the same point in time, whereby as temperature decreased, the proportion of piglets resting in contact with the sow increased (*P* = 0.005; Figure 5). Day of life also influenced piglet-sow proximity, where piglets were most likely to be resting in contact with the sow earlier in life (*P* < 0.001; Figure 6). Pairwise comparisons showed there was a higher proportion of piglets in contact with the sow on day 0 compared to all other days, with 62% (95% CI = 59 to 65%) of piglets resting in physical contact with the sow on the first day of life, compared to 54% (95% CI = 51 to 57%) on day 1, 45% (95% CI = 42 to 48%) on day 2, 41% (95% CI = 38 to 44%) on day 3, and 40% (95% CI = 37 to 43%) on day 4. There was also a significant effect of time of day on piglet-sow proximity (*P* = 0.030), where the odds of piglets resting in contact with the sow at 1900h was 1.5× greater than at 1200h (*P* = 0.002). The litter’s weight classification did not affect the proportion of piglets resting in contact with the sow (*P* = 0.859).

### Shivering and huddling and hut temperature

There was a significant effect of hut temperature on the presence of piglets shivering from 1200 to 2300h over the first four days of life (*P* < 0.001), whereby as temperature decreased, shivering increased. For every 1°C increase in temperature, the likelihood of shivering decreased by 15% (OR = 0.85, 95% CI = 10 to 20%). There was also a significant impact of day of age on shivering behaviour when piglets were resting, where the likelihood of shivering reduced with age, with a significant difference in shivering between day 0 and 3 (*P* < 0.001; Figure 6). On the first day of life, the probability of shivering was 27% (95% CI = 18 to 35%), compared to 18% (95% CI = 11 to 25%) on day 1, 17% (95% CI = 10 to 23%) on day 2, 11% (95% CI = 6 to 16%) on day 3 and 14% (95% CI = 8 to 20%) on day 4. There was a significant effect of the time of day, with piglets increasingly likely to shiver as it got later from 1200 to 2300h (*P* < 0.001). There was no significant effect of litter weight on shivering presence (*P* > 0.05).

For every 1°C increase in hut temperature, there was a 19% decrease in the presence of the litter huddling in one group (OR = 0.81, 95% CI = 15% to 24; *P* < 0.001).There was no significant effect of day of age on huddling behaviour, with the probability of huddling being high across all days (day 0, 85% [95% CI = 80 to 90%]; day 1, 82% [95% CI = 77 to 86%]; day 2, 84% [95% CI = 80 to 89%]; day 3, 80% [95% CI = 75 to 84%]; and day 4, 82% [95% CI = 77 to 87%]). There was also no significant effect of weight classification or time of day on the presence of piglets huddling (*P* > 0.05).

### Piglet survival and piglet-sow proximity

There was no evidence that piglet-sow proximity from days 1 to 4 of life affected piglet survival. This included no detected effect of the average proportion of piglets resting in contact with the sow on the number of piglet deaths from days 1 to 4 (OR = 0.99; *P* = 0.989; N_Litters_ = 57), or the number of deaths on day 0 (OR = 0.08; *P* = 0.301; N_Litters_ = 58). There was also no significant effect of litter weight or sow parity on models fitting the effect of piglet-sow proximity on piglet survival (*P* > 0.05).

## Discussion

This research investigated how decreasing farrowing hut temperatures influence piglet behaviour and survival during winter. We hypothesised that in colder huts, piglets would engage in more thermoregulatory behaviours – particularly increased contact with the sow – increasing the risk of death. This hypothesis was partly supported: while there was no relationship between piglet survival and seeking physical contact from the sow, there was strong evidence that piglets in colder huts were more likely to die early in life, and they also shivered, huddled, and engaged in more physical contact with the sow.

### Hut climate, thermoregulatory behaviours and piglet survival

The present research showed that piglets in outdoor farrowing systems were more likely to die during winter if the climate inside the farrowing hut was not optimal, particularly in the first days of life. Cecil *et al.* (2025) found that 17–21°C with a 35°C creep area is optimal for sows and piglets in indoor systems, but in the present research minimum hut temperatures were up to 18°C lower than this bottom range and piglets had no supplementary heating. It is therefore not surprising that as hut temperature decreased piglet mortalities increased during the critical period of survival on day 0 and days 1–4. These findings are in contrast to a Danish study by Schild *et al.* (2019) who found that hut climate had no effect on early liveborn piglet mortality. However, these findings are consistent with prior research looking at outdoor pig production in different climates, such as Scandinavia (Rangstrup-Christensen *et al.* 2018b), the UK (KilBride *et al.* 2014), and the US (Honeyman & Roush 2002). Additionally, when examining seasonal variation in piglet mortality, Berger *et al.* (1997) and Randolph *et al.* (2005) found increased mortality over early winter in outdoor litters in France and England, respectively. Notably, many of these climates are likely to be significantly colder than the present research where the ambient temperature ranged from 1.5 to 22.2°C during the study period, based on recordings from the closest weather station approximately 16 km from the farm. This demonstrates that cold stress is still a considerable welfare concern for piglets even in Australia where the temperature is more moderate in winter.

From 5 to 12 days of life, there was no effect of hut temperature on piglet mortality. This is likely because piglets still alive by this age are better equipped to handle the environment around them, post the critical period of mortality (Marchant *et al.* 2000). Homeothermy progressively increases over the first four days of life, as demonstrated by increased rectal temperature, and thus by day 5, hut temperature is unlikely to affect mortality (De *et al.* 2024).

Interestingly, there was a significant effect of minimum hut temperature, but not average hut temperature, on piglet mortalities before postnatal litter management on the first day of life. Metabolic studies have identified a significant drop in piglet rectal temperature immediately after birth by at least 2°C, which rebounds within 24 h to an appropriate temperature (Mount 1967; Lossec *et al.* 1998). In Lossec and colleague’s study (1998), experimentally induced hypothermic conditions were set at 14°C. In the present research the median average daily temperature was above this, while the minimum temperature was below. This may explain why a significant relationship was only detected for minimum temperature on the first day of life, highlighting the potential impact of short periods of cold exposure on piglet survival within the first 24 h of farrowing. Further, it highlights the importance of measuring minimum instead of average hut temperature.

In the present research, piglet mortality was examined by considering all causes of death together. Ascertaining accurate causes of piglet mortality remains highly challenging (Edwards *et al.* 1994; Edwards & Baxter 2015). In the present study, overlay was predominantly the recorded cause of death, however, it is possible some piglets died of causes like chilling and were subsequently overlain, leading to the cause of death determined by the farm being inaccurately recorded as overlay. For instance, despite evidence that piglets were in a challenging thermal environment, there were only two cases where the cause of death on the first day of life was recorded as exposure. More detailed information regarding the specific causes of death would be of value to better understand the relationships between farrowing hut climate, piglet behaviour and piglet mortality. Nonetheless, despite the limitations identified, it is clear piglet mortality increases in outdoor pig production systems when farrowing huts are too cold.

As hut temperature decreased, piglets were more likely to rest in physical contact with the sow. This supports the widely established notion that contact with the sow is a thermoregulatory mechanism (Weary *et al.* 1996; Vasdal *et al.* 2009). Piglet-sow proximity decreased with age, which is consistent with prior studies by Jahoui *et al.* (2024), and Blackshaw and Hagelsø (1990). Although lower hut temperatures were associated with reduced survival and increased piglet-sow proximity from days 0 to 4, there was no evidence that piglet-sow proximity early in life affected piglet survival. Therefore, while piglets may spend more time in contact with the sow when the hut is colder, this does not appear to increase the risk of overlays, or death from other causes. This refutes the idea of the “danger zone”, illustrated by Pokorná *et al.* (2008) as the area within one piglet length of the sow immediately before she lies down. The Pokorná *et al.* study however examined piglets in intensive systems with the addition of a heated creep area, so piglets may be clustered in proximity to the sow for thermoregulatory purposes less often. Marchant *et al.* (2001) found that proximity to the sow is not inherently a risk factor if the piglets are huddled together, and it is when piglets are scattered and “disorganised” that they are more at risk. This is likely because when the piglets are all in one group, it is easier for the sow to distinguish their location and avoid them when changing postures. In the present research, piglets were huddling in one group most of the time, which supports this argument.

Alongside an increased likelihood of resting in contact with the sow, there was evidence that piglets were more likely to be shivering and huddling in one single group as hut temperature reduced. This aligns with previous research identifying shivering thermogenesis as a key thermoregulatory response in piglets (Berthon *et al.* 1994; Lossec *et al.* 1998) and the influence of temperature on huddling in the first week of life (Newberry & Wood-Gush 1988; Conrad *et al.* 2022) as key thermoregulatory responses in piglets. As expected, piglets were less likely to shiver as they got older. In line with these findings, Berthon *et al.* (1994) found that shivering intensity declines over the first five days of life, despite cold-induced heat production remaining relatively constant. This is likely due to improved thermogenic efficiency because of increased metabolic activity (Berthon *et al.* 1994). There was also a significant effect of time of day on shivering in the present research, where the odds of shivering trended towards decreasing overnight, however, these should be interpreted with caution, as the video footage quality was lower overnight. This was due to the reduced contrast and brightness and may impair the ability to accurately identify shivering. While shivering has been proposed as a practical on-farm indicator of hypothermia, it is important to note that not all hypothermic piglets display this behaviour, as shivering occurs when core body temperature is still above 34°C (Herpin *et al.* 2002). Therefore, the piglets most at risk, at a later stage of hypothermia may be missed upon initial observation (Tabuaciri *et al.* 2012).

There was no effect of day of age, piglet weight, or time of day on huddling behaviours, and the overall incidence was high, with > 80% of litters observed huddling across days 0 to 4. Supporting this, Vasdal *et al.* (2009) reported up to 80% of piglets regularly huddling regardless of temperature during the early post-natal period. Taken together, the high incidence of sow contact, shivering, and huddling indicate thermal conditions during winter in typical Australia outdoor production conditions may pose welfare challenges for piglets. Improving thermal conditions through door flaps to retain heat (Lucas *et al.* 2025) or supplementary heat sources could improve piglet thermal comfort as well as survival, benefiting animal welfare and farm performance.

### Effects of weight and sow parity on piglet survival

Previous research has shown smaller piglets to be considerably less likely to survive the first week of life compared to larger piglets (Vasdal *et al.* 2009; Marchant *et al.* 2000; Muns *et al.* 2016; Nuntapaitoon *et al.* 2018), and the present research identified the first four days of life as a critical period for survival of small piglets. Small piglets are more prone to being overlain, which is often attributed to small piglets spending more time spent in close proximity to the sow, due to an increased need to access milk and thermoregulate (Weary *et al.* 1996; Herpin *et al.* 2002). However, the present research found no effect of the litter’s weight classification on the likelihood of resting in physical contact with the sow. These findings should be interpreted with caution, as weight categories were estimated by stockpeople which may have introduced variability in the classification across litters. There may be effects of weight that are observed at an intra-litter level, as prior research has identified individual piglet weight to be a major risk factor for mortality (Zotti *et al.* 2017). Although small piglets are at an increased risk of dying from overlays, there are often a range of co-factors and co-morbidities that when combined with decreased vigour and energy reserves, lead to piglets being less able to avoid overlay. These co-factors and co-morbidities include starvation, acute disease and hypothermia (Herpin *et al.* 2002; Chidgey *et al.* 2022). Supporting this, Kobek-Kjeldager *et al.* (2023) found that when examining fatal overlay events in outdoor housing, one-third of piglets were already exhibited low vitality. Therefore, the observed increased incidence of deaths of small piglets may be due to their reduced ability to avoid the sow, intensified by these co-factors, which reduce their movement and responsiveness, as opposed to their proximity to the sow.

Sow parity has previously been shown to affect piglet mortality. While past studies found that as parity increased, so did mortality in outdoor production systems (Rangstrup-Christensen *et al.* 2018a,b), the present research found first parity sows to have the highest incidence of pre-weaning mortality, compared to parity 2–4 and 5–10 sows. However, there were very few primiparous sows included in the present research (5/200), so these conclusions should be interpreted cautiously. Additionally, differences in climatic and genetic conditions, including the prolificacy of sows, may also explain differences between studies.

## Animal welfare implications and conclusion

This research demonstrated a clear relationship between decreased outdoor farrowing hut temperature during winter and increased piglet mortality. Colder huts were also associated with piglets engaging in more thermoregulatory behaviours like shivering, huddling, and resting in contact with the sow, suggesting poor thermal comfort. Although piglets sought more sow contact as hut temperature reduced, there was no evidence this behaviour was linked with increased piglet mortality. This implies mortality risk in outdoor farrowing huts is not driven by increased proximity to the sow, but perhaps instead by reduced mobility in cold-stressed piglets, limiting their ability to avoid crushing. Overall, the high prevalence of sow contact, shivering and huddling, along with reduced survival in colder huts, indicates cold stress is a considerable welfare concern for piglets in outdoor farrowing systems even in relatively moderate winter conditions common to Australia. There is opportunity to improve the thermal conditions of outdoor farrowing huts through modifications that increase heat retention or provide supplementary heating. Doing so would enhance piglet thermal comfort and survival, delivering benefits for both animal welfare and farm productivity.
